# Cardiac SNARE Expression in Health and Disease

**DOI:** 10.3389/fendo.2019.00881

**Published:** 2019-12-19

**Authors:** Peter R. T. Bowman, Godfrey L. Smith, Gwyn W. Gould

**Affiliations:** ^1^Henry Wellcome Laboratory of Cell Biology, College of Medical, Veterinary and Life Sciences, Institute of Molecular Cell and Systems Biology, University of Glasgow, Glasgow, United Kingdom; ^2^College of Medical, Veterinary and Life Sciences, Institute of Cardiovascular and Medical Sciences, University of Glasgow, Glasgow, United Kingdom

**Keywords:** diabetes, cardiomyopathy, SNARE proteins, insulin resistance, GLUT4

## Abstract

SNARE proteins are integral to intracellular vesicular trafficking, which in turn is the process underlying the regulated expression of substrate transporters such as the glucose transporter GLUT4 at the cell surface of insulin target tissues. Impaired insulin stimulated GLUT4 trafficking is associated with reduced cardiac function in many disease states, most notably diabetes. Despite this, our understanding of the expression and regulation of SNARE proteins in cardiac tissue and how these may change in diabetes is limited. Here we characterize the array of SNARE proteins expressed in cardiac tissue, and quantify the levels of expression of VAMP2, SNAP23, and Syntaxin4—key proteins involved in insulin-stimulated GLUT4 translocation. We examined SNARE protein levels in cardiac tissue from two rodent models of insulin resistance, *db/db* mice and high-fat fed mice, and show alterations in patterns of expression are evident. Such changes may have implications for cardiac function.

## Introduction

Effective regulation of metabolism is essential in all cell types in order to ensure that ATP generation requirements are met. This is particularly important within highly energetic organs such as the heart, where the contractile action of cardiomyocytes must be continually fuelled in order to maintain the pumping of approximately 5 liters of oxygen and nutrient rich blood into and around the systemic circulation every minute. It is also vital that the heart exhibits metabolic flexibility in order to adapt its contractile output in response to increased demands e.g., during exercise. Normal cardiac metabolism is characterized by predominant use of fatty acids as a metabolic substrate, with a relatively lower utilization of glucose (1). This is logical as fat is a more abundant and energy rich fuel source, making it ideal for scenarios where sustained moderate levels of ATP are required. However, several cardiac disease states are partly defined (and potentially caused) by deficits in glucose uptake and metabolism.

The predominant type 2 diabetic phenotype is characterized by peripheral insulin resistance, whereby insulin no longer effectively stimulates the uptake of glucose into muscle and adipose tissue. The mechanism underlying this condition is multifactorial, but is strongly linked to obesity (2). This physiological action of insulin is important in maintaining glycaemic control post-prandially, and indeed diabetes is diagnosed by an elevation in fasting blood glucose (or circulating glycated hemoglobin) above clinically defined thresholds. In a contracting working heart preparation from a diabetic mouse model, radiolabelled substrates revealed an impairment of insulin stimulated glycolysis and glucose oxidation and increased basal fatty acid utilization (associated with reduced myocardial efficiency) and an impaired ability of insulin to reduce this (3). Additionally, increasing the rate of fatty acid delivery to control hearts resulted in reduced glycolysis and glucose oxidation, enhanced fatty acid utilization, and reduced cardiac performance (3). This demonstrates the importance of insulin-stimulated glucose uptake to fatty acid metabolism (and vice versa) and therefore cardiac efficiency and performance and exemplifies potential defects in these systems in diseases such as diabetes.

Cardiovascular disease has long been established to be a leading cause of death in the diabetic population, in part attributable to the high rate of vascular disease that occurs on account of sustained excessive glucose (and fat) in the bloodstream (4). However, there is also a direct pathological effect of diabetes upon cardiac function, characterized by initial diastolic dysfunction prior to structural remodeling and progression to heart failure, termed diabetic cardiomyopathy (5–7). There is strong evidence that metabolic impairments such as the intramyocellular accumulation of lipids in the heart and associated cardiac insulin resistance may be critical early factors in the progression of this condition (8–10). Most notably, in a mouse model of diabetic cardiomyopathy, overexpression of the insulin sensitive glucose transporter GLUT4 restored aberrant cardiac metabolic and contractile function to values observed in controls (11, 12). Additionally, insulin resistance/glycaemic control has also been demonstrated to be of prognostic significance in human post-myocardial infarction patients (13–15), with an experimental rat model identifying the onset of cardiac (not systemic) insulin resistance to be correlated with adverse recovery/remodeling (16).

It has been established that general concepts derived from other insulin sensitive cell types are applicable to the heart, for example that GLUT4 is the functionally predominant glucose transporter (17). Under basal conditions the majority of GLUT4 is not located at the cell surface but rather distributed between the general endosomal recycling pathway and a depot of specialized GLUT4 storage vesicles (GSVs) that can be rapidly mobilized to the cell surface in response to activation of the insulin receptor (18). Both the sorting of GLUT4 through different intracellular compartments and the fusion of GSVs with the plasma membrane (PM) requires the action of specific SNARE proteins (19). In skeletal muscle and fat tissue, the SNARE proteins that regulate GSV fusion with the PM are VAMP2, SNAP23, and Syntaxin 4, whereas Syntaxin 6 and 16 mediate sequestration of GLUT4 into pools of GSVs at the trans Golgi network (20–25).

Numerous studies have used a variety of techniques (including knock down or expression of dominant mutant proteins) to disrupt the function of SNARE proteins in the insulin sensitive cell model 3T3-L1 adipocytes and demonstrated a clear inhibitory effect upon insulin dependent GLUT4 trafficking and glucose uptake, with the precise defect depending on the protein targeted (26–30). Furthermore, *in vivo* examination of mice over or under-expressing Syntaxin 4 identified improved/impaired skeletal muscle insulin stimulated glucose uptake and therefore also whole body glucose tolerance, attributed to corresponding effects on insulin stimulated GLUT4 translocation (31, 32). To underline this strong association between SNARE proteins, GLUT4 trafficking, and glycaemic control, even manipulation of the expression of ancillary proteins such as Munc18c and Doc2b that regulate SNARE interactions has significant consequences on GLUT4-PM integration and glucose uptake (33, 34). Insulin-stimulated GLUT4 translocation in peripheral tissues thus exhibits a considerable degree of mechanistic overlap, with studies emphasizing the role of Syntaxin 4 and SNAP23 in fat and muscle cells, and transgenic mice clearly establishing that levels of expression of these SNAREs correlate with whole-body glycaemic control (31, 32).

The involvement of SNAREs in multiple steps of GLUT4 trafficking make them an intriguing potential target in the context of disease. There are many theories relating to insulin resistance, such as inhibition of proximal insulin signaling via either lipid mediated activation of protein kinase C (35, 36) or altered release of adipocytokines from expanded and inflamed adipose tissue (37, 38). However, there is also evidence from rodent models correlating altered SNARE protein (Syntaxin4, Syntaxin6, VAMP2, VAMP3, SNAP23, Munc18) expression or localization with skeletal muscle and adipose insulin resistance (31, 32, 39–41). Additionally, in human type 2 diabetic patients enhanced syntaxin 8 expression in adipose tissue was significantly associated with reduced GLUT4 expression and impaired whole body glucose tolerance (42). While it is not clear from these studies whether alterations in SNARE protein levels are causal or adaptive changes, initial studies from 2 independent insulin resistant models indicate that targeting SNARE proteins may be a viable strategy to restore insulin stimulated GLUT4 trafficking and improve metabolic outcomes (43–45).

Any attempt to investigate these initial observations in the heart is constrained by very limited prior characterization of SNARE protein expression in cardiomyocytes, let alone how they may interact or be of functional importance. This is in contrast to considerable investigation in adipose and skeletal muscle. The only specific prior attempt to identify the expression and involvement of SNAREs in cardiac GLUT4 trafficking was performed in a mouse atrial cell line (HL-1) and restricted to assessment of all VAMP (v-SNARE) isoforms (46). Expression of VAMP2/3/4/5/7 (but not VAMP1 or 8) protein was detected, and this was confirmed in lysate generated from mouse heart. Targeted silencing of each isoform revealed a role for VAMP2 and VAMP5 in the insulin stimulated appearance of GLUT4-myc at the PM. However, separate work with rodent cardiomyocytes identified the expression of VAMP1/2/3/4/5/8 (but not VAMP7) mRNA transcripts (47). Whilst this study did not investigate the expression of all VAMPs beyond the use of RT-PCR, this data highlights that even within this limited field contradictions have emerged regarding the expression of VAMP1/7/8 in the heart. It is possible that this is in part due to the use of isolated cardiomyocytes (47). Whilst isolation of cells prior to analysis reduces interference in the sample from other subpopulations of cardiac cells, if maintained in culture dedifferentiation may start to occur which could impact protein expression.

Therefore, the aim of this work was to characterize the expression of a wide range of SNARE isoforms within primary adult cardiac tissue. Furthermore, it was assessed if the expression of these proteins (in addition to GLUT4) was altered in 2 different diabetic mouse models. This study is the first step toward uncovering the role of different SNAREs in insulin stimulated cardiac GLUT4 trafficking and is of clinical relevance due to the association of cardiac insulin resistance with diabetic cardiomyopathy and myocardial infarction. SNAREs are also important in both non-insulin stimulated (e.g., contraction mediated) GLUT4 trafficking and the trafficking of the fatty acid transporter CD36 (46). Therefore, this work is valuable and foundational in the context of the regulation of cardiac metabolism in general.

## Results and Discussion

### Quantification of VAMP2, Syntaxin 4, and SNAP23 in Cardiac Lysates

**Figure 1:**
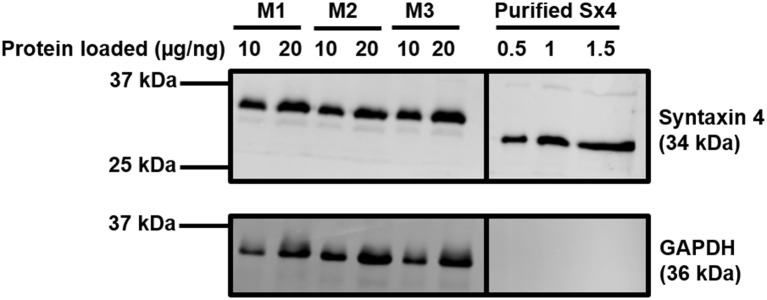
Quantification of Syntaxin 4 expression in the heart. Protein lysates were generated from 20-week old male mouse cardiac tissue and subjected to SDS-PAGE and immunoblotting alongside purified recombinant Sx4. Lysates were incubated with antibodies probing for the expression of Syntaxin 4 and GAPDH (loading control) as shown. Protein loaded refers to 10 or 20 μg of protein for the M1-M3 mouse cardiomyocyte samples or 0.5–1.5 ng of purified Syntaxin 4 protein (lacking the transmembrane domain and thus migrating faster than endogenous, full length Syntaxin4), as indicated. M1-3 indicates biologically independent mouse cardiac samples, where each sample is composed of lysate generated from 2 to 3 individual hearts. The approximate positions of molecular weight markers are indicated. Borders indicate where images have been cropped for presentation purposes only. Data from a representative immunoblot is shown, quantified in Table 1.

In adipocytes and muscle, the SNARE proteins associated with GSV-PM fusion are VAMP2, Syntaxin 4, and SNAP23 (26, 27, 30). We first sought to quantify expression of these SNAREs in rodent cardiac lysates. Recombinant SNARE proteins were expressed and purified from bacteria and used as standards in quantitative immunoblotting to compare to the signal obtained from mouse cardiac samples. An example of this technical approach is shown in Figure 1 for quantification of Syntaxin 4 expression, with quantification of all 3 SNAREs reported in Table 1. Comparative values displayed for 3T3-L1 adipocytes were obtained from previously published work using a similar approach (48). This technique was also performed with a pooled lysate generated from cardiac tissue derived from 3 individual rat hearts. The mean values obtained closely matched those of the mouse samples (1.45 × 10^12^ copies per mg SNAP23; 6.82 × 10^11^ copies per mg Sx4; 3.05 × 10^11^ copies per mg VAMP2).

**Table 1:** Quantification of SNARE protein expression in primary mouse cardiac tissue.

**Sample**	**SNAP23 Copies/mg**	**Syntaxin 4 Copies/mg**	**VAMP2 Copies/mg**
Mouse Heart	2.07 × 10^12^ (± 5.66 × 10^11^)	7.26 × 10^11^ (± 2.67 × 10^10^)	3.23 × 10^11^ (± 3.56 × 10^10^)
3T3-L1 Adipocytes	5.28 × 10^12^	1.72 × 10^12^	2.28 × 10^12^

*Densitometry was used to quantify Syntaxin 4, SNAP23, and VAMP2 expression from known amounts of primary cardiac tissue and purified recombinant protein, within immunoblots such as that depicted in Figure 1. Values for recombinant proteins were used to generate a standard curve that allowed estimation of the absolute amount of each protein in a given amount of total cardiac lysate. This value is expressed in copies per mg of total lysate and is the mean (±S.E.M.) of at least 3 biologically independent replicate samples. Values from 3T3–L1 adipocytes are those reported in Hickson et al. (48)*.

In 3T3-L1 adipocytes there are approximately 2–3 fold more SNAP23 molecules in comparison to VAMP2 and Syntaxin 4 (48). It is acknowledged that within this study our technical approach will be associated with a margin of error, however this phenomenon may not be specific to adipocytes as a similar relative expression of key SNARE proteins is recorded here from two sources of primary cardiomyocytes. The reason underlying this observation requires further study but most plausibly may reflect the involvement of SNAP23 in multiple other fusion events relevant to other biological processes. Additionally, this data provides one possible explanation as to why 3T3-L1 adipocytes display a greater fold insulin response compared to primary cardiomyocytes. Despite expressing less GLUT4 (49), 3T3-L1 adipocytes express abundantly more of each SNARE protein thereby generating a more favorable ratio between the availability of transporters and fusion machinery. However, it must also be acknowledged that the heart tends to display an elevated basal rate of glucose uptake in order to support contractile demands (50), which would therefore reduce any fold insulin response. These data also confirm that, like 3T3-L1 adipocytes, Syntaxin 4 and VAMP2 are expressed at broadly similar levels on a per cell basis.

### SNARE Expression in Diabetic Mouse Models

Insulin resistance may have an important role in the development of limitations in cardiac contractile performance in diabetic individuals. Accordingly, given their involvement in multiple steps of GLUT4 trafficking, SNARE proteins may be implicated in this clinically relevant pathophysiological setting. It is therefore necessary to characterize the array of SNARE trafficking machinery present in primary cardiac tissue in order to probe known functions of these proteins (in other cell types) in the heart, and novel physiological roles or interactions specific to cardiac function. It is challenging to obtain a significant amount of human myocardial samples for biochemical investigation, particularly from specific patient populations and appropriately matched controls. In order to circumnavigate this issue, many animal models of disease have been developed in the field of cardiac physiology. In the context of diabetes one of the most widely utilized and characterized models is the *db/db* mouse, which has a leptin receptor mutation that results in obesity and blood parameters mimicking the human diabetic state (51). Importantly, this model not only displays systemic insulin resistance, but also cardiomyocyte-specific insulin resistance and reduced cardiac contractility (recorded at the level of the whole heart) (52, 53), making it an ideal tool for investigation of DCM.

**Figure 2:**
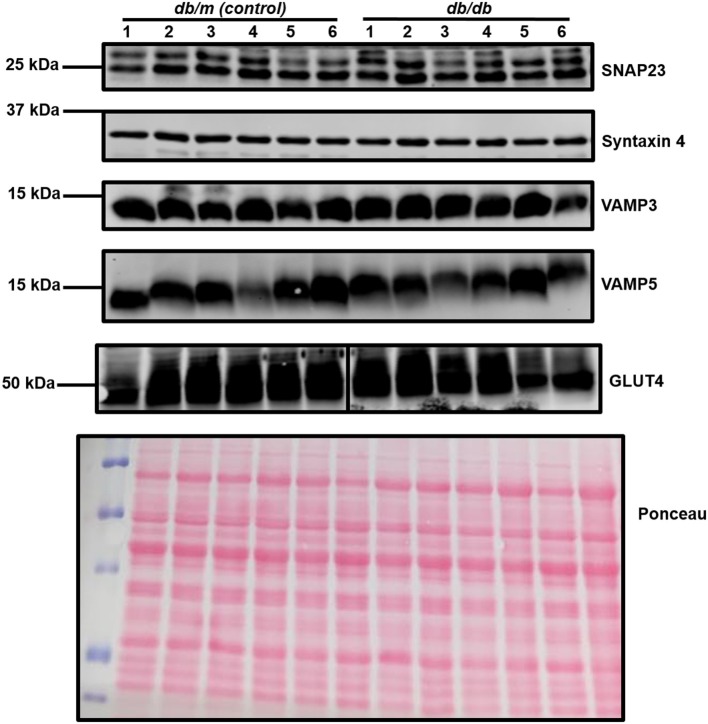
Cardiac SNARE expression in *db/db* and *db/m* mice. Protein lysates were generated from *db/db* and control mouse hearts and subjected to SDS-PAGE and immunoblotting, probing for the expression of numerous SNARE proteins and GLUT4. Representative immunoblots for selected proteins only are shown. A representative Ponceau S staining of a membrane is displayed, which was used as a loading control. For each sample, 40 μg of protein lysate was loaded. Each sample (1–6 on figure) is composed of lysate generated from a separate heart.

Within this study, cardiac segments from 6 *db/db* and control (*db/m*) mice were lysed and probed for the expression of a broad range of SNARE proteins and GLUT4 via immunoblotting. A typical dataset is displayed in Figure 2. Consistent with previous analysis of cardiac protein expression from this diabetic mouse model (53, 54), GLUT4 expression was found to be significantly (*P* < 0.05) reduced in *db/db* lysates. We observed clear and consistent immunoblot signals for Sx2, 4, 5, 8, and 16, SNAP23, 29 and 47, and VAMP2, 3, 4, 5, and 8 (Supplementary Table 1), but as shown for a subset in Figure 2, no significant differences between *db/m* and *db/db* mice were observed for any SNARE proteins. In contrast to Schwenk et al. (46) but consistent with Peters et al. (47), we were unable to detect VAMP7 in primary cardiac tissue yet obtained a clear signal for VAMP8. However, in support of Schwenk et al. (46) but in opposition to Peters et al. (47), we were unable to detect VAMP1.

The *db/db* mouse is considered to be a good representation of the established human diabetic phenotype. However, there are legitimate questions regarding the translational relevance of this model. Whilst there is almost certainly a genetic component in many cases of type 2 diabetes (55), more commonly lifestyle factors—e.g., food intake and activity levels—that determine overall net energy balance are recognized as key factors in diabetes prevalence. In particular, obesity greatly increases the risk of an individual developing diabetes (2). Therefore, high fat diet (HFD) based interventions in rodents have been developed extensively. This produces a less severe diabetic phenotype typically analogous to a pre-diabetic state (56), however still captures reduced cardiac contractility in the context of insulin resistance (57). It is likely that the mechanisms underpinning complex phenotypes such as insulin resistance and cardiomyopathy may be multifactorial and vary with disease progression. Therefore, we examined SNARE protein expression in a mouse HFD model.

**Figure 3:**
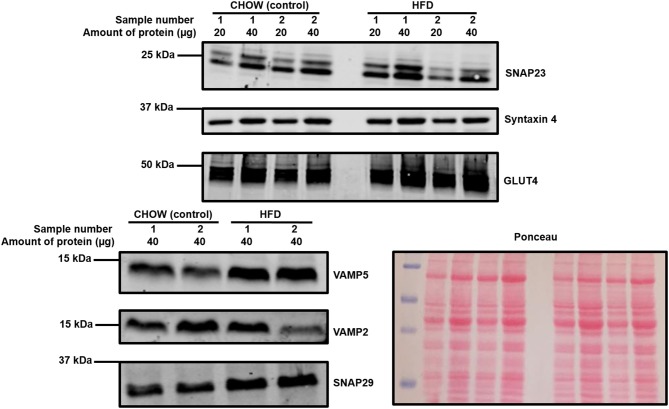
Cardiac SNARE expression in HFD fed and control mice. Protein lysates were generated from HFD and control mouse hearts and subjected to SDS-PAGE and immunoblotting, probing for the expression of SNARE proteins and GLUT4. Representative immunoblots for selected proteins are shown together with Ponceau S staining, which was used as a loading control. Each sample is composed of lysates generated from 2 to 4 individual hearts.

As can be observed in Figure 3 and Table 2, an almost identical range of SNARE proteins were detected in this second independent mouse cohort as observed in Figure 2. Due to limitations in sample availability, lysates from 2 to 4 individual hearts were pooled in order to generate each of the 4 samples displayed, which reduced the statistical power of subsequent analysis. With this limitation in mind, there was no impact of the pro-diabetic phenotype upon GLUT4 expression. However, statistically significant elevations in VAMP5 (78% increase) and SNAP29 (68%) expression were detected. It appeared as though VAMP2 expression may have been reduced in at least one experimental group, however this effect was not found to be statistically significant.

**Table 2:** Quantification of SNARE protein expression in HFD primary cardiac lysates.

**Protein**	**Detected?**	**Difference?**	**Protein**	**Detected?**	**Difference?**
SNAP23	Yes	No	Syntaxin 16	Yes	No
SNAP29	Yes	Yes, significantly (*P* = 0.04) increased in HFD lysates by 68%	GLUT4	Yes	No
SNAP47	Yes	No	VAMP1	No	N/A
Syntaxin 2	Yes	No	VAMP2	Yes	30% lower expression in HFD lysates, difference was not significant (*P* = 0.33)
Syntaxin 3	Yes	No	VAMP3	Yes	No
Syntaxin 4	Yes	No	VAMP4	Yes	No
Syntaxin 5	Yes	No	VAMP5	Yes	Yes, significantly (*P* = 0.02) increased in HFD lysates by 78%
Syntaxin 6	Yes	No	VAMP7	No	N/A
Syntaxin 7	No	N/A	VAMP8	Yes	No
Syntaxin 8	Yes	No			

*Immunoblotting was used to detect the presence or absence of a range of proteins in cardiac lysates generated from HFD and CHOW control mice. Where a clear and reproducible difference was observed between groups, densitometry was used to quantify expression relative to total protein (from Ponceau stained images) and an unpaired Student's t-test was used to assess statistical significance (N = 2). The level of significance was set at P = 0.05*.

The samples used in Figures 2, 3 were obtained from sections of frozen tissue. Therefore, measurements could not be obtained that could confirm or refute that these lysates were from insulin resistant hearts. However, we note that the *db/db* mouse is a reliable and well characterized model and our observation of reduced GLUT4 expression supports the disease phenotype being evident in these samples. Importantly, published data from the same control and HFD mice cohort confirms that samples used here were from mice that displayed significant weight gain and a trend toward delayed systemic glucose clearance during a glucose tolerance test compared to controls—indicative of reduced insulin sensitivity (58).

We have shown a range of SNARE proteins are expressed in primary cardiac samples. This exceeds the machinery we may expect to be required for the regulated trafficking of substrate transporters such as GLUT4 or CD36. Indeed, the heart is known to secrete vasoactive natriuretic peptides in order to regulate blood pressure and therefore cardiac loading, and recent work has also highlighted the emerging role that numerous cardiokines may have in modulating both cardiac remodeling in response to sustained pathophysiological stress through auto/paracrine signaling and also remote physiological functions through endocrine actions (59). The secretion of these signaling proteins will be reliant upon the action of SNAREs. Our study will provide the platform necessary to relate SNARE function to specific physiological actions, and therefore ultimately investigate any potential role in disease. Additionally, this study provides clarity where there had previously been conflicting data regarding the expression of VAMP1/7/8. Obtaining identical results for these proteins from 2 independent mouse lines provides confidence in these findings.

Interestingly, in the HFD model SNAP29 and VAMP5 expression were elevated relative to control samples. VAMP5 has previously been linked to GLUT4 trafficking, but only in a cardiomyocyte cell line model (46). In contrast, the functional role of SNAP29 is not clear, particularly in the heart. Given that impaired GLUT4 trafficking underlies insulin resistance, it is unclear why expression of a related protein would remain elevated beyond any initial compensatory period. It is interesting to note however that elevated SNARE protein levels were observed in skeletal muscle of the ZDF rat model of diabetes, which were reversed by treatment with thiazolidinediones (39). Hence, there is a precedent for elevated SNARE expression in insulin resistance. In contrast to the study of skeletal muscle insulin resistance which saw elevations in Sx4 and VAMP2, here we report elevated VAMP5 in cardiac tissue from HFD mice. VAMP5 has also been implicated in the regulation of contraction-mediated glucose uptake in cardiomyocytes (46) and thus our observation may reflect a functional distinction between skeletal muscles and cardiomyocytes. Elevated VAMP5 may change (or reflect) the contractile profile of the heart in the early stages of diabetes prior to the onset of cardiomyopathy. It might be speculated that enhanced contraction is used to compensate for reduced insulin mediated glucose uptake at certain times. Alternatively, the observed changes may relate to the trafficking of another target, perhaps a fatty acid transporter. Enhanced cardiac lipid content is one of several factors in the onset of insulin resistance, but this can only occur with increased trafficking of the relevant transporters to the cell membrane. Further work will be required to answer these questions and to define whether the changes observed in cardiac SNARE proteins in disease models are causal or adaptive responses.

## Conclusion

This study has detailed, for the first time, the expression of a wide range of SNARE proteins in primary cardiac samples. This foundational work paves the way for future studies investigating the interactions and roles of these proteins in several different aspects of cardiac physiology, both under normal working conditions and in disease.

## Materials and Methods

### Primary Cardiac Samples

All primary cardiac tissues used during this study were kind gifts from colleagues at the University of Glasgow; procedures were undertaken in accordance with the United Kingdom Animals (Scientific Procedures) Act of 1986 and conform to the Guide for the Care and Use of Laboratory Animals published by the US National Institutes of Health and approved by the Glasgow University Ethical Review Board. In all cases tissues were received as sections of myocardium that had been snap frozen either at −80°C or in liquid nitrogen. Key details pertaining to each sample are listed in Table 3. All of the samples gifted were from male animals, therefore it is acknowledged that there may be sex specific differences in the reported findings that require further investigation.

**Table 3:** Information regarding primary cardiac samples.

**Sample**	**Key details**	**Gifted by**
*Db/db* and *db/m* mice	13–15-week-old male mice either hetero or homozygous for a mutation in the leptin receptor gene	Dr. Augusto Montezano
High Fat Diet fed and control mice	Approximately 20-week-old male mice fed standard CHOW diet for 8 weeks postnatally prior to switching to a diet whereby 60% of calories were obtained from fat for the next 12 weeks (or maintained on CHOW as control)	Dr. Anna White

### Generation of Lysates

The procedure for generation of cardiac lysates was the same regardless of the origin of the tissue. First of all, a large section of myocardium was excised from each heart. These sections were generated in the same way by the same experimenter, without prior separation into distinct anatomical regions. The vast majority of myocardium is ventricular tissue, and therefore any large section of cardiac tissue will almost certainly originate predominantly from this area. Each section was placed within a plastic 10 cm dish on ice in RIPA buffer at a ratio of approximately 1-part tissue to 5 parts buffer. The tissue was then manually diced with a sterile scalpel blade into sections as small as possible, prior to further mechanical lysis via a Dounce style tissue grinder. Samples were placed on ice for a further 20 min, prior to repetition of this mechanical lysis technique. The lysates were then centrifuged at 17,500 g for 15 min at 4°C. The supernatant was then collected, and the pellet was discarded. Prior to immunoblotting the protein concentration of each sample was calculated via micro BCA assay.

### Generation and Quantification of Purified Protein

Recombinant purified protein samples were generated as described previously by Sadler et al. (60). First of all, plasmids encoding sequences for Syntaxin 4, SNAP23, and VAMP2 tagged with glutathione S-transferase (GST) but in the case of Sx4 and VAMP2 lacking their transmembrane domains to increase expression were transformed into BL-21 *E.coli* and subsequently amplified in serial cultures, using ampicillin (100 μg/mL) resistance as a selection marker. Once in the optimal growth range and volume, recombinant protein production was induced via Isopropyl B-D-1-thiogalactopyranoside (0.5 mM) incubation overnight at 22°C. The following day cells were lysed, and target proteins were extracted through incubation of the lysate for 2 h with glutathione beads. Thereafter, the beads were washed with PBS and then the samples were eluted. Prior to use within this study, the GST tags were cleaved from the proteins. Quantification of purified protein concentration was performed by running samples through SDS-PAGE against known amounts of BSA and then performing Coomassie staining in order to visualize all protein bands, as described by Sadler et al. (60). Gels were scanned and then densitometry was performed in order to generate a protein standard curve with BSA signals, which was then used to estimate the concentration of each purified SNARE sample.

### SDS-PAGE and Immunoblotting

Samples were defrosted on ice, combined 1:1 with 2x Laemmli sample buffer, and then heated to 65°C for 10 min. Homemade polyacrylamide gels of the appropriate percentage were cast and then lysates were loaded in order to achieve the desired (often equal) amount of protein across different samples. These were then separated via gel electrophoresis and subsequently subjected to immunoblotting as described previously by Sadler et al. (60). Densitometry was performed via Image Studio Lite in order to quantify the generated images. Either GAPDH expression or Ponceau S staining was used in order to assess total protein loading.

### Antibodies

Anti-GLUT1 (#652) and anti-GLUT4 (#654) were from AbCam (Cambridge, United Kingdom). Anti-GAPDH (#4300) was from Ambion (Foster city, California, USA). Anti-VAMP1 (#104002), anti-VAMP2 (#104202), anti-VAMP3 (#43080), anti-VAMP4 (#136002), anti-VAMP5 (#176003), anti-VAMP7 (#232003), anti-VAMP8 (#104302), anti-SNAP23 (#111202), anti-SNAP29 (#111303), anti-SNAP47 (#111403), anti-syntaxin2 (#110022), anti-syntaxin3 (#110032), anti-syntaxin4 (#110042), anti-syntaxin5 (#110053), anti-syntaxin7 (#110072), anti-syntaxin8 (#110083), and anti-syntaxin16 (#110162) were from Synaptic Systems (Goettingen, Germany). Anti-syntaxin6 (#610635) was from BD Biosciences (Franklin lakes, New Jersey, USA). Fluorescent secondary antibodies were from LI-COR Biosciences (Lincoln, Nebraska, USA).

### Statistical Analysis

Statistical testing was performed with GraphPad Prism 7. Where appropriate, this consisted of an unpaired *t*-test, assessing the difference in protein expression between one of the experimental groups and relevant control. The level of significance was set at *P* = 0.05.

## Data Availability Statement

The datasets generated for this study are available on request to the corresponding author.

## Ethics Statement

The animal study was reviewed and approved by Glasgow University Ethical Review Board.

## Author Contributions

PB designed and performed the experiments, analyzed the data, prepared the figures, and wrote the first draft. GS and GG conceived the study, obtained funding, designed the study, and supervised the laboratory work. GS and GG edited the manuscript.

### Conflict of Interest

The authors declare that the research was conducted in the absence of any commercial or financial relationships that could be construed as a potential conflict of interest.
