# Supplementary material for: Cardiac SNARE Expression in Health and Disease
Source: Front Endocrinol (Lausanne). 2019 Dec 19;10:881. doi: 10.3389/fendo.2019.00881 (PMC6930865; doi:10.3389/fendo.2019.00881)
Supplement: Supplementary file 1 [file Table_1.docx]

**Supplemental Table 1. Quantification of SNARE protein expression in *db/db* primary cardiac lysates.** Immunoblotting was used to detect the presence or absence of a range of proteins in cardiac lysates generated from *db/db* and *db/m* control mice. Where a clear and reproducible difference was observed between groups, densitometry was used to quantify expression relative to total protein (from Ponceau stained images) and an unpaired Student’s t-test was used to assess statistical significance (N=6). The level of significance was set at P=0.05.

| Protein | Detected? | Difference? | Protein | Detected? | Difference? |
| --- | --- | --- | --- | --- | --- |
| SNAP23 | Yes | No | **GLUT4** | Yes | Yes, significantly (P=0.004) decreased in *db/db* lysates by 45% (+/- 8%) |
| SNAP29 | Yes | No | **VAMP1** | No | N/A |
| SNAP47 | Yes | No | **VAMP2** | Yes | No |
| Syntaxin 2 | Yes | No | **VAMP3** | Yes | No |
| Syntaxin 3 | No | N/A | **VAMP4** | Yes | No |
| Syntaxin 4 | Yes | No | **VAMP5** | Yes | No |
| Syntaxin 5 | Yes | No | **VAMP7** | No | N/A |
| Syntaxin 8 | Yes | No | **VAMP8** | Yes | No |
| Syntaxin 16 | Yes | No |  |  |  |
